# Rapid Tumor Targeting of Renal-Clearable ZW800-1 Conjugate for Efficient Photothermal Cancer Therapy

**DOI:** 10.3390/biomedicines9091151

**Published:** 2021-09-03

**Authors:** Min Ho Park, Gayoung Jo, Bo Young Lee, Eun Jeong Kim, Hoon Hyun

**Affiliations:** 1 Department of Surgery, Chonnam National University Medical School and Hwasun Hospital, Hwasun 58128, Korea; mhpark@jnu.ac.kr (M.H.P.); angeleunei@naver.com (E.J.K.); 2 Department of Biomedical Sciences, Chonnam National University Medical School, Hwasun 58128, Korea; jky6213@naver.com (G.J.); 0000by@naver.com (B.Y.L.); 3 BioMedical Sciences Graduate Program (BMSGP), Chonnam National University, Hwasun 58128, Korea

**Keywords:** zwitterionic fluorophores, ZW800-1, photothermal therapy, tumor targeting, renal clearance

## Abstract

The combination of near-infrared (NIR) fluorophores and photothermal therapy (PTT) provides a new opportunity for safe and effective cancer treatment. However, the precise molecular design of functional NIR fluorophores with desired properties, such as high tumor targetability and low nonspecific uptake, remains challenging. In this study, a renal-clearable NIR fluorophore conjugate with high tumor targetability was developed for efficient photothermal cancer therapy. The isoniazid (INH)–ZW800-1 conjugate (INH–ZW) was synthesized by conjugating an antibiotic drug, INH, with a well-known zwitterionic NIR fluorophore, ZW800-1, to improve in vivo performance and fluorescence-guided cancer phototherapy. INH–ZW not only showed rapid tumor accumulation without nonspecific tissue/organ uptake within 1 h after the injection but also generated thermal energy to induce cancer cell death under NIR laser irradiation. Compared with previously reported ZW800-1 conjugates, INH–ZW preserved the ideal biodistribution of ZW800-1 and facilitated improved tumor targeting and PTT. Together, these results demonstrate that the INH–ZW conjugate has great potential to serve as an effective PTT agent capable of rapid tumor targeting and high renal clearance, with excellent photothermal efficacy.

## 1. Introduction

Near-infrared (NIR) fluorophores have great potential in biomedical applications for image-guided cancer surgery and photothermal therapy (PTT), with distinct advantages, including reduced tissue autofluorescence, targeted tumor imaging, and high photothermal conversion capabilities [1,2,3,4,5]. Ideal NIR fluorophores, as PTT agents, should selectively accumulate in tumor tissue and clear rapidly from normal tissue for safe and accurate cancer treatment. To achieve a high tumor targetability and an excellent photothermal performance, NIR fluorophores armed with carboxyl or amine groups are typically employed for covalent conjugation with various kinds of specific ligands, including small molecules [6,7], peptides [8,9], and proteins [10,11].

Several types of cyanine-based fluorophores (e.g., IRDye800CW, Cy5.5, MHI-148, and ZW800-1) have been previously developed to conjugate with tumor-targeting ligands, such as cyclic RGD peptide, folic acid, and sorbitol, for in vivo tumor imaging [7,8,12,13,14]. However, the efficiency of tumor targeting still remains challenging because the targetability of ligands can be altered by the physicochemical properties of fluorophores after conjugation [8]. Among these conjugatable fluorophores, the zwitterionic NIR fluorophore ZW800-1 displays no serum binding and exhibits ultralow nonspecific tissue/organ uptake, rapid renal excretion from the body, and remarkable optical properties, thereby allowing target-specific imaging after conjugation with ligands [8,15,16].

Despite the superior in vivo performance of ZW800-1, considerable limitations remain for the selection of ligands depending on the structural characteristics. According to previous studies, unbalanced surface charges of the ZW800-1 conjugates result in unexpected nonspecific uptake, decreased renal excretion, or reduced targetability of ligands during systemic circulation [9,17]. Thus, the correct combination of ZW800-1 and a tumor-targeting ligand could play a critical role in the preferential tumor accumulation without nonspecific uptake and the fast renal excretion to prevent potential cytotoxicity of the conjugate.

Recently, several studies reported the use of MHI-148 conjugates based on isoniazid (INH), a small molecule inhibitor of monoamine oxidase A that is clinically used as the first-line anti-tuberculosis medicine, for targeted cancer imaging and therapy [18,19]. Although the MHI-148 and INH conjugate showed significant antitumor efficacy, there is a fundamental limitation of such NIR fluorophores, namely, persistent nonspecific binding, uptake, and retention in normal organs, including the heart, lungs, liver, spleen, and kidneys [18,19]. In this study, we developed a renal-clearable PTT agent, INH–ZW, by conjugating the zwitterionic NIR fluorophore ZW800-1 with the antibiotic drug INH to improve the in vivo performance and fluorescence-guided photothermal cancer therapy. The INH–ZW conjugate preserved the ideal biodistribution of ZW800-1 and compensated for tumor targetability, thereby acting as a bifunctional phototherapeutic agent.

## 2. Experimental Section

### 2.1. Conjugation of Isoniazid to the ZW800-1 NIR Fluorophore (INH–ZW)

All chemicals and solvents were of American Chemical Society grade or high-performance liquid chromatography (HPLC) purity. The starting materials were purchased from Sigma-Aldrich (St. Louis, MO, USA) and were used without purification. The ZW800-1 NIR fluorophore was synthesized as described previously [15,16]. Isoniazid (1.1 mg, 7.9 μM), also known as isonicotinylhydrazine (INH), was conjugated to ZW800-1 (5 mg, 5.3 μM) in the presence of 4-(4,6-dimethoxy-1,3,5-triazin-2-yl)-4-methylmorpholinium chloride (DMT-MM; 3 mg, 10 µmol) in DMSO (5 mL) at room temperature for 12 h. The crude mixture was separated using a preparative HPLC system equipped with a 150 mL PrepLC fluid handling unit, a manual injector (Rheodyne 7725i; Thermo Scientific, Waltham, MA, USA), and a 2487 dual wavelength absorbance detector (Waters, Milford, MA, USA). The molecular weight of the purified INH–ZW conjugate was verified with mass spectroscopy using an ultra-performance liquid chromatography (UPLC, Waters) device equipped with micrOTOF-Q II (Bruker, Ettlingen, Germany).

### 2.2. Optical and Physicochemical Property Analyses

All the optical measurements were performed in phosphate-buffered saline (PBS), pH 7.4. The absorption spectrum of INH–ZW was measured using a fiber optic FLAME absorbance and fluorescence (200–1025 nm) spectrometer (Ocean Optics, Dunedin, FL, USA). The molar extinction coefficient was calculated using the Beer–Lambert equation. The fluorescence emission spectrum of the INH–ZW conjugate was analyzed using a SPARK^®^ 10M microplate reader (Tecan, Männedorf, Switzerland) at an excitation wavelength of 700 nm and emission wavelengths ranging from 750 to 900 nm. In silico calculations of the partition coefficient (log*D* at pH 7.4) and topological polar surface area (TPSA) were performed using the Marvin and JChem calculator plugins (ChemAxon, Budapest, Hungary).

### 2.3. In Vitro Cell Binding and NIR Fluorescence Microscopy

The human colorectal adenocarcinoma cell line HT-29 and mouse embryonic fibroblast cell line NIH/3T3 were obtained from the American Type Culture Collection (ATCC, Manassas, VA, USA). Cells were maintained in Roswell Park Memorial Institute (RPMI) 1640 or Dulbecco’s Modified Eagle Medium (DMEM) media (Gibco BRL, Paisley, UK) supplemented with a 10% fetal bovine serum (FBS, Gibco BRL) and an antibiotic-antimycotic solution (100 units/mL penicillin, 100 μg/mL streptomycin, and 0.25 μg/mL amphotericin B; Welgene, Daegu, South Korea) in a humidified 5% CO_2_ atmosphere at 37 °C. When the cells reached a confluence of approximately 50%, they were rinsed twice with PBS and the INH–ZW or ZW800-1 NIR fluorophore was added to each well at various concentrations in the range of 2–50 μM; the cells were incubated for 24 h at 37 °C. They were then gently washed with PBS. NIR fluorescence imaging was performed using a four-filter set on a Nikon Eclipse Ti-U inverted microscope system. The microscope was equipped with a 100 W halogen lamp, NIR-compatible optics, and an NIR-compatible 10X Plan Fluor objective lens (Nikon, Seoul, South Korea). Image acquisition and analysis were performed using the NIS-Elements Basic Research software (Nikon). NIR filter sets containing 750 ± 25 nm excitation filters, 785 nm dichroic mirrors, and 810 ± 20 nm emission filters were used to detect the NIR fluorescence signals in the cells. All the NIR fluorescence images were acquired at identical exposure times and normalized.

### 2.4. In Vitro Cytotoxicity Assay

Cell toxicity and proliferation were evaluated using the alamarBlue™ (Thermo Scientific, Waltham, MA, USA) assay. The HT-29 cells were seeded onto 96-well plates (1 × 10^4^ cells per well). To determine cytotoxicity depending on the concentration, the cancer cells were treated with the INH–ZW conjugate (2, 10, 25, and 50 μM) for 1 h and cultured for 24 h after treatment. At each assay time point, the incubation cell medium was replaced with 100 μL of fresh medium, and 10 μL of the alamarBlue solution was directly added to each 100 μL well; the plates were then incubated for 4 h at 37 °C in a humidified 5% CO_2_ incubator. Finally, the 96-well plates were placed in a microplate reader (SPARK^®^ 10M, Tecan) to measure the absorption intensity at 570 nm and the fluorescence intensity at 590 nm. Cell viability was calculated using the following formula (*A* is the average absorbance): cell viability (%) = (*A*_sample_ − *A*_blank_)/(*A*_control_ − *A*_blank_) × 100.

### 2.5. HT-29 Xenograft Mouse Model

Animal care, experiments, and euthanasia were performed in accordance with protocols approved by the Chonnam National University Animal Research Committee (CNU IACUC-H-2017-64). Adult (6-week-old) male NCRNU mice weighing approximately 25 g (*n* = 3 independent experiments) were purchased from OrientBio (Seongnam, South Korea). HT-29 cancer cells were harvested and suspended in 100 μL of PBS before being subcutaneously injected in the right flank of each mouse (1 × 10^6^ cells per mouse). When tumor sizes reached about 1 cm in diameter, INH–ZW and ZW800-1 were administered intravenously. Animals were euthanized and imaged over a certain period.

### 2.6. In Vivo Biodistribution and Tumor Imaging

In vivo NIR fluorescence imaging was performed using an FOBI imaging system (NeoScience, Suwon, South Korea). Mice were sacrificed 1 and 4 h after injection, and their main organs (heart, lungs, liver, pancreas, spleen, kidneys, duodenum, and intestine) were collected and imaged to evaluate the time-dependent biodistribution of INH–ZW. The fluorescence intensities of the tumors and organs were analyzed using ImageJ version 1.45q (National Institutes of Health, Bethesda, MD, USA). All images were identically normalized for all conditions.

### 2.7. In Vivo Photothermal Therapeutic Efficacy

HT-29 tumor mice were intravenously injected with PBS, ZW800-1, or INH–ZW and anaesthetized after 1 h. The tumors were irradiated with a laser (1.1 W/cm^2^, *λ* = 808 nm) for 5 min. Temperature changes in tumors were monitored using an FLIR^®^ thermal imager (FLIR Systems, Wilsonville, OR, USA). Data were recorded with a step size of 1 min throughout the whole laser irradiation process. After 24 h post irradiation, tumors were excised from the treated mice for subsequent histological analysis with hematoxylin and eosin (H&E) staining. To confirm the in vivo antitumor effect, the macroscopic morphology of each group was observed at determined time intervals for 9 days. Tumor volume (V) was calculated using the following formula: V = 0.5 × longest diameter × (shortest diameter)^2^.

### 2.8. Statistical Analysis

A one-way analysis of variance (ANOVA) and a Tukey’s multiple comparison test were performed. Differences were considered statistically significant at *p* < 0.05. The results are presented as mean ± standard deviation (S.D.) and curve fitting was performed using Prism software version 4.0a (GraphPad, San Diego, CA, USA).

### 2.9. Histological Analysis and NIR Fluorescence Microscopy

Resected tumors were preserved for H&E staining and microscopic assessment. The samples were fixed in 2% paraformaldehyde and flash-frozen in an optimal cutting temperature (OCT) compound using liquid nitrogen. Frozen samples were cryosectioned (10 µm thick slides), observed by fluorescence microscopy, and then stained with H&E. Histological imaging was performed on a Nikon Eclipse Ti-U inverted microscope system. Image acquisition and analysis were performed using the NIS-Elements Basic Research software (Nikon). All the NIR fluorescence images were acquired at identical exposure times and normalized.

## 3. Results and Discussion

### 3.1. Synthesis and Characterization of INH–ZW Conjugate

**Figure 1:**
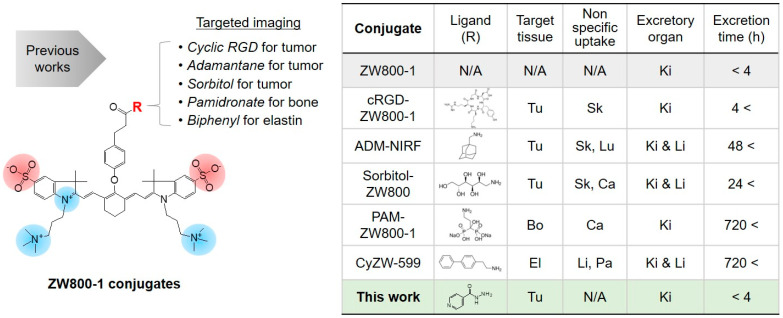
Structures of ZW800-1 [15] and its conjugates with various targeting ligands (e.g., cRGD-ZW800-1 [8], ADM-NIRF [20], Sorbitol-ZW800 [7], PAM-ZW800-1 [17], and CyZW-599 [21]) reported previously for NIR fluorescence imaging. Comparison of in vivo performance characteristics from the current study and previous studies in terms of targetability, nonspecific tissue/organ uptake, and excretion time. Abbreviations: Bo, bone; Ca, cartilage; El, elastin; Ki, kidneys; Li, liver; Lu, lungs; Pa, pancreas; Sk, skin; Tu, tumor; N/A, not applicable.

Initially, the zwitterionic NIR fluorophore ZW800-1 was designed by Choi et al. to have a balanced net surface charge, resulting in ultralow nonspecific uptake and rapid renal clearance [15]. Furthermore, the carboxyl group in the structure of ZW800-1 enabled conjugation with various targeting ligands such as cyclic RGD peptide [8], adamantane [20], and sorbitol [7] for tumor imaging, pamidronate [17] for bone imaging, and 2-(4-biphenyl)ethylamine [21] for elastin imaging. As summarized in Figure 1, the ZW800-1 conjugates achieved target-specific imaging; however, the structural changes after conjugation could result in unexpected uptake by tissues/organs, including the skin, lungs, liver, cartilage, and pancreas, and delayed excretion from the body. In this study, we designed a rapid renal-clearable INH–ZW conjugate, by combining ZW800-1 with the small-molecule INH to improve tumor targetability, for use in effective photothermal cancer treatment.

**Figure 2:**
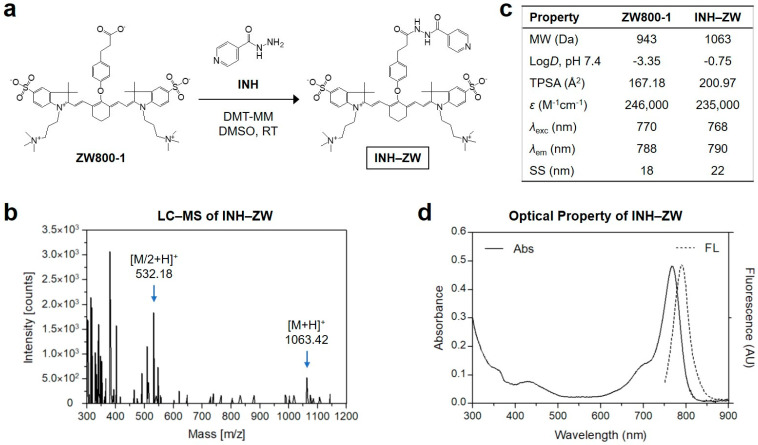
(**a**) Synthesis scheme; (**b**) mass spectrum; (**c**) physicochemical and optical properties; (**d**) absorbance and fluorescence emission spectra of the INH–ZW conjugate. Optical measurements were performed in PBS at pH 7.4. In silico calculations of log*D* at pH 7.4 and the topological polar surface area (TPSA) were performed using the Marvin and JChem calculator plugins (ChemAxon, Budapest, Hungary).

The INH molecule was covalently conjugated to the ZW800-1 fluorophore through amide bond formation via a condensation reaction in the presence of a coupling agent (Figure 2a). The INH–ZW conjugate was purified using a preparative HPLC system, and analyzed via liquid chromatography–mass spectrometry (LC–MS) to verify the successful synthesis, for further use in in vitro and in vivo studies (Figure 2b). Additionally, the in silico prediction of the physicochemical properties, including hydrophobicity (log*D*) and polarity (TPSA), of the INH–ZW conjugate was performed using JChem (ChemAxon) (Figure 2c). INH–ZW showed similar optical properties to those of ZW800-1. Importantly, the increased hydrophobicity and polarity of the INH–ZW conjugate may have played critical roles in the improved tumor targeting. The maximum absorption and fluorescence emission spectra of the INH–ZW conjugate in the NIR region were measured at 768 and 790 nm, respectively (Figure 2d). This suggests that the INH–ZW conjugate can be used for photothermal cancer treatment when combined with an 808 nm NIR laser.

### 3.2. In Vitro Cancer Cell Binding and Cytotoxicity

**Figure 3:**
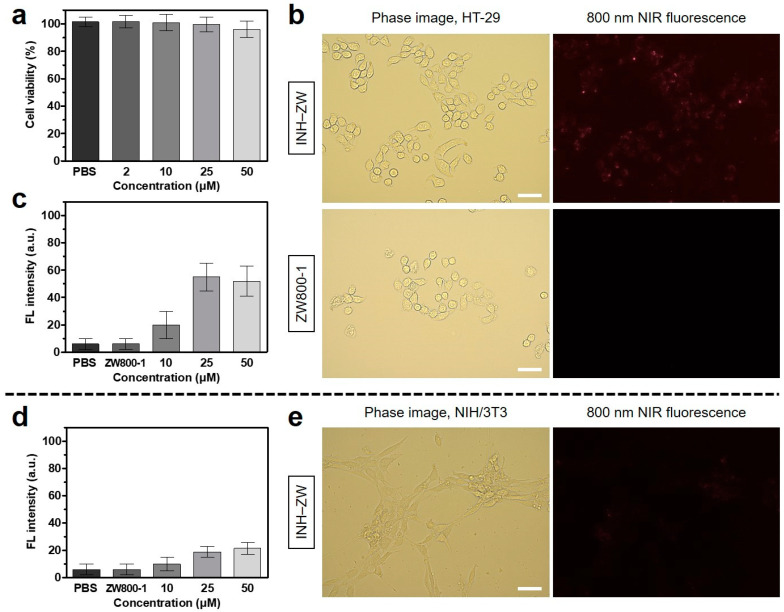
(**a**) Cell viability assay of the INH–ZW conjugate using HT-29 cancer cells. The percentage cytotoxicity was determined after 24 h of treatment with various concentrations of the INH–ZW conjugate. (**b**) Live cancer cell binding of the INH–ZW conjugate and ZW800-1 in HT-29 cells. Relative fluorescence intensities in (**c**) HT-29 and (**d**) NIH/3T3 cells 24 h after treatment with various concentrations of the INH–ZW conjugate or 25 µM ZW800-1. (**e**) Live normal cell binding of the INH–ZW conjugate in NIH/3T3 cells. The phase contrast and NIR fluorescence images of the cell line were obtained using 25 µM INH–ZW or ZW800-1. Data are expressed as the mean ± S.D. of the three independent experiments. Images are representative of three independent experiments. All NIR fluorescence images have identical exposure times and normalization. Scale bars = 100 μm.

To confirm the cellular binding and cytocompatibility of the INH–ZW conjugate, the HT-29 cancer cell line was used for in vitro assessment. To estimate cytotoxicity, the alamarBlue assay was performed to determine the relative viability of HT-29 cancer cells after incubation with the INH–ZW conjugate at various concentrations (2, 10, 25, and 50 μM). Interestingly, no significant cytotoxicity to the HT-29 cancer cells was observed, even at the high INH–ZW concentration of 50 μM (Figure 3a). This suggests that no significant toxicity was induced by the INH–ZW conjugate. Additionally, the intracellular distribution of the INH–ZW conjugate was observed with NIR fluorescence microscopy after 24 h of incubation in HT-29 cancer cells. Although fluorescence signals of INH–ZW, which corresponded to intracellular localization, were detected in the cancer cells 24 h post treatment, the observed fluorescence intensity was low and slightly higher than that of cells treated with ZW800-1 (Figure 3b). The fluorescence signals of the INH–ZW conjugate in the cancer cells were barely evident at high concentrations of 25–50 μM, and they were unmeasurable in the concentration range of 2–10 μM (Figure 3c). Moreover, the binding specificity of INH–ZW on normal cells was investigated using the fibroblast cell line NIH/3T3. As shown in Figure 3d,e, the INH–ZW conjugate exhibited relatively weak binding affinity in normal cells compared with HT-29 cancer cells. This demonstrates that the INH moiety would provide more favorable binding to cancer cells. In accordance with Lipinski’s rule, the theoretical TPSA value (200.97 Å^2^) of INH–ZW was greater than 140 Å^2^, which tends to be poor at permeating cell membranes [22]. These results indicate that the zwitterionic property of INH–ZW may have reduced the cellular uptake of the INH moiety, which is consistent with the good cytocompatibility of the INH–ZW conjugate.

### 3.3. Time-Dependent In Vivo Tumor Imaging and Biodistribution

**Figure 4:**
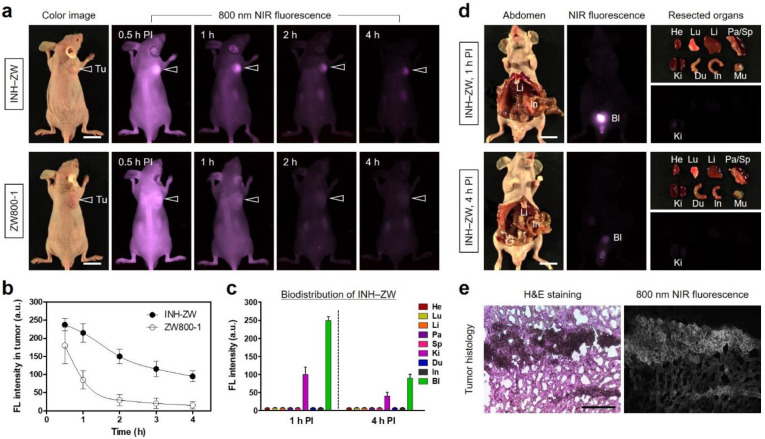
In vivo HT-29 tumor targeting efficiency and biodistribution of the INH–ZW conjugate. (**a**) NIR fluorescence imaging 4 h after injection of INH–ZW and ZW800-1. (**b**) Time-dependent fluorescence intensities at the tumor sites targeted by INH–ZW and ZW800-1. (**c**) Quantitative fluorescence analysis of intraoperative dissected organs 1 and 4 h after injection of INH–ZW. (**d**) Biodistribution and resected organs imaged 1 and 4 h after injection of INH–ZW. Tumor-bearing mice were intravenously injected with 10 nmol of INH–ZW or ZW800-1 and imaged for 4 h. (**e**) H&E staining and NIR fluorescence imaging of the resected tumor tissues 1 h after injection of INH–ZW. The tumor sites are indicated by arrowheads. Abbreviations: Bl, bladder; Du, duodenum; He, heart; In, intestines; Ki, kidneys; Li, liver; Lu, lungs; Mu, muscle; Pa, pancreas; Sp, spleen; Tu, tumor; PI, post injection. Scale bars = 1 cm (white bars) and 300 μm (black bar). Images are representative of three independent experiments. All NIR fluorescence images had identical exposure times and normalization. Data are expressed as the mean ± S.D. of three independent experiments.

The in vivo tumor targetability and biodistribution of INH–ZW were investigated in an HT-29 xenograft mouse model. To determine the tumor accumulation of the INH–ZW conjugate compared to that of ZW800-1, tumor-bearing mice were intravenously administered 10 nmol of INH–ZW or ZW800-1 and monitored using a real-time NIR fluorescence imaging system (Figure 4a). Based on the time-dependent NIR fluorescence images, the high fluorescence intensity at the tumor site treated with the INH–ZW conjugate was maintained until 1 h after injection, while the fluorescence signal of tumor treated with ZW800-1 rapidly decreased without tumor-specific accumulation until 4 h after injection (Figure 4b). In terms of the tumor-to-background ratio, the optimal time point for conducting PTT was determined to be 1 h after INH–ZW administration, to avoid unnecessary damage to adjacent tissues. This finding demonstrated that the INH moiety of the INH–ZW conjugate played a key role in the improved tumor targeting and would allow this conjugate to be applied for photothermal cancer treatment. Additionally, the biodistribution of INH–ZW was confirmed by analyzing and comparing the fluorescence signals from the major organs excised from mice 1 and 4 h after injection (Figure 4c). Importantly, the INH–ZW conjugate showed no significant organ/tissue uptake 1 h after injection owing to the rapid renal excretion, which is a well-known characteristic of the ZW800-1 NIR fluorophore. The renal-clearable INH–ZW conjugate was dominantly detected in the bladder, with strong fluorescence 1 h after injection, and it was completely eliminated from the body after only 4 h (Figure 4d). Compared with previously reported ZW800-1 conjugates, the INH–ZW conjugate preserved the ideal biodistribution of ZW800-1 and facilitated the improved tumor imaging within 1 h of injection. Since the INH–ZW conjugate could be accumulated in tumors at 1 h post injection, we further performed histological analysis combined with NIR fluorescence microscopy. The NIR fluorescence microscopic image revealed the existence of INH–ZW in the tumor tissue (Figure 4e).

### 3.4. In Vitro and In Vivo Photothermal Effects

**Figure 5:**
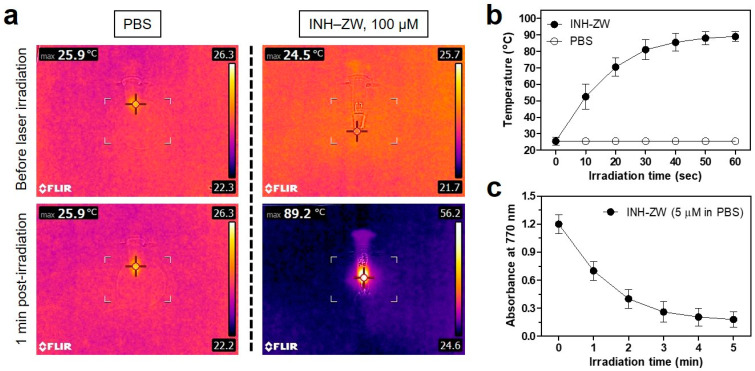
(**a**) In vitro thermal images of the INH–ZW solution (10 μg/100 μL in PBS; 100 μM concentration is equivalent to a single dose of 0.4 mg/kg) and PBS alone (100 μL) exposed to an 808 nm laser (1.1 W/cm^2^) for 1 min. The maximum temperature was automatically recorded using an infrared thermal camera as a function of the irradiation time. (**b**) Temperature changes in the solutions in each sample were monitored during the 1 min of laser irradiation. (**c**) Photostability of the INH–ZW solutions under laser irradiation. The absorbance changes in 5 μM INH–ZW solutions were measured at 770 nm during the 5 min of laser irradiation. Data are expressed as the mean ± S.D. of three independent experiments.

The photothermal properties of the INH–ZW conjugate (10 μg/100 μL PBS; 100 μM as a single dose of 0.4 mg/kg) and PBS (100 μL) solutions were investigated through a 1 min exposure to 808 nm laser irradiation (1.1 W/cm^2^). Temperature changes were recorded at intervals of 10 s using an FLIR^®^ thermal imager. Under NIR laser irradiation, the temperature of the INH–ZW solution rapidly increased from 24.5 to 89.2 °C over 1 min, while the PBS solution showed no temperature change (Figure 5a). The temperature of the INH–ZW solution remarkably increased to ~80 °C during the first 30 s of laser irradiation and reached up to ~90 °C during the next 30 s of irradiation (Figure 5b). This demonstrated that the INH–ZW conjugate had a highly efficient photothermal conversion capability; thus, it is a promising PTT agent. In addition, the photothermal conversion efficiency (*η*) of the INH–ZW conjugate was calculated to be 34.1%, which was based on the previous method [23]. This value is comparable to that of a sorbitol–ZW800 conjugate (32.6%) reported previously [14]. To confirm the photostability of the INH–ZW conjugate, the absorbance of the INH–ZW solution was repeatedly measured at 770 nm after every 1 min of laser irradiation. As expected, the absorption values of the INH–ZW solution gradually decreased during the 5 min of laser irradiation, which indicated that the heptamethine cyanine core of ZW800-1 degraded after exhibiting light-to-heat conversion (Figure 5c).

**Figure 6:**
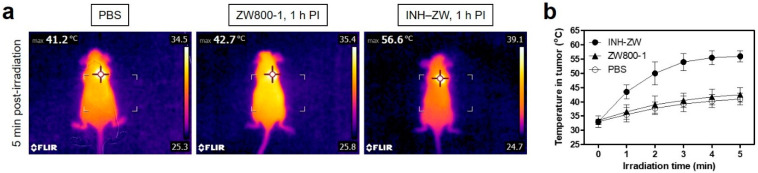
(**a**) Whole-body thermal images of tumor-bearing mice 1 h after injection of PBS, ZW800-1, or INH–ZW upon exposure to 808 nm laser irradiation (1.1 W/cm^2^) for 5 min. (**b**) Temperature changes at the tumor sites in each treatment group were monitored during the 5 min of 808 nm laser irradiation. Data are expressed as the mean ± S.D. of three independent experiments.

Furthermore, the PTT capability of the INH–ZW conjugate in vivo was investigated using an HT-29 tumor-bearing mouse model. Mice were intravenously injected with INH–ZW, ZW800-1, or PBS 1 h before laser irradiation, and tumor sites were subsequently exposed to 808 nm laser irradiation at 1.1 W/cm^2^ for 5 min. The temperature of tumors treated with INH–ZW rapidly increased up to ~56 °C, while the temperatures of tumors injected with PBS or ZW800-1 reached only ~42 °C during the 5 min of laser irradiation (Figure 6a). Importantly, tumor temperatures in the INH–ZW treatment group peaked after 3 min of laser irradiation and remained at ~56 °C until the final 2 min of laser irradiation, which was sufficient to induce the apoptosis of cancer cells directly (Figure 6b). This result indicates that the INH–ZW conjugate can be utilized as a tumor-targeted PTT agent for effective cancer treatment.

### 3.5. In Vivo Photothermal Therapeutic Efficacy

**Figure 7:**
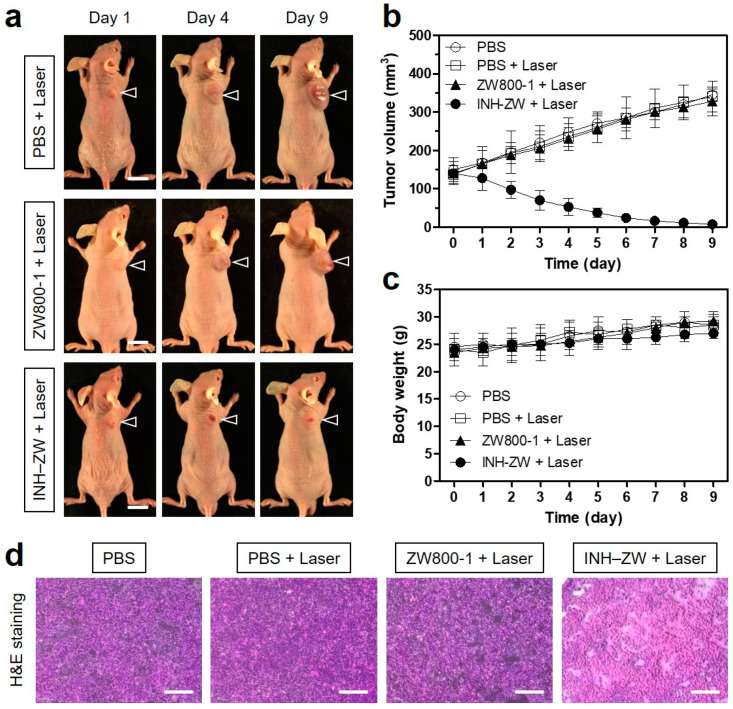
In vivo NIR phototherapeutic efficacy. (**a**) Representative photos of tumor size changes in HT-29 tumor-bearing mice for 9 days after different treatments. The laser groups were treated with 1 h post injections of PBS, ZW800-1, and INH–ZW, followed by 808 nm laser irradiation (1.1 W/cm^2^) for 5 min. The tumor sites are indicated by arrowheads. Scale bars = 1 cm. (**b**) Tumor growth rates and (**c**) body weights of each treatment group were monitored for 9 days. Data are expressed as the mean ± S.D. of three independent experiments. (**d**) Tumor sections stained with H&E from each group after 24 h of different treatments. Scale bars = 100 μm.

To evaluate the in vivo phototherapeutic effect of INH–ZW, the tumor sizes were monitored continuously for 9 days after the NIR laser treatment (Figure 7a). Without the PTT agent INH–ZW, the tumors in mice treated with PBS or ZW800-1 followed by laser irradiation showed similar growth rates to those of the tumors of mice treated with PBS alone, indicating that there were no therapeutic effect and tissue damage caused by only laser irradiation. With the PTT agent, tumor growth in the INH–ZW and laser-treated mice was effectively inhibited (Figure 7b). This demonstrated that the combination of INH–ZW and NIR laser irradiation could completely ablate the tumor without recurrence during the course of the treatment. Additionally, no signs of body weight loss and mortality were evident in the INH–ZW treatment group, which demonstrated the high biosafety of this treatment (Figure 7c). Furthermore, the tumors harvested from each group 24 h after different treatments were stained with H&E to confirm the histological changes (Figure 7d). In comparison to the PBS and ZW800-1 groups, which showed no cell damage, most cells in the tumors treated with INH–ZW and laser irradiation were apparently necrotic, which was indicated by a reduced cell number and shrunken nuclei. This result demonstrates that the INH–ZW conjugate generated a mean tumor temperature of ~56 °C that was sufficiently higher than the threshold temperature (~42 °C) in the PBS and ZW800-1 groups to induce cell apoptosis and necrosis. These findings indicate that the INH–ZW conjugate could be successfully used as a bifunctional agent for tumor-targeted imaging and effective photothermal cancer treatment.

## 4. Conclusions

In this study, we synthesized a renal-clearable ZW800-1 conjugate (INH–ZW) that could specifically target a tumor, and applied it in real-time NIR fluorescence imaging and photothermal cancer therapy. The INH–ZW conjugate exhibited not only rapid tumor accumulation but also a high renal clearance within 1 h of injection. Moreover, the molecular characteristics of INH–ZW enabled the generation of thermal energy under 808 nm laser irradiation, leading to the complete ablation of the tumor with no evident side effects. The bifunctional INH–ZW conjugate preserved the significant advantages of the zwitterionic NIR fluorophore ZW800-1 and compensated for tumor targetability, which demonstrated its suitability for fluorescence-guided cancer phototherapy. In this regard, the optimal design of the ZW800-1 conjugates can provide a principled approach for improving in vivo performance with low nonspecific uptake, which is an important consideration for the clinical use of contrast agents. In conclusion, INH–ZW shows great promise as a phototherapeutic agent for tumor-targeted imaging and photothermal therapy in various biomedical applications.

## Data Availability

Not applicable.
